# Consumption of spices and ethnic contamination in the daily diet of Italians - consumers’ preferences and modification of eating habits

**DOI:** 10.1186/s42779-021-00082-8

**Published:** 2021-06-05

**Authors:** Stefania Chironi, Simona Bacarella, Luca Altamore, Pietro Columba, Marzia Ingrassia

**Affiliations:** 1 Department of Agricultural, Food and Forest Sciences, Università degli Studi di Palermo, Viale delle Scienze, Building 4, Palermo, Italy; 2 Department of Economics, Business and Statistics, Università degli Studi di Palermo Viale delle Scienze, Building 13, Palermo, Italy

**Keywords:** Spices, Traditional foods, International food products, Consumption habits, Food contamination, Food tradition

## Abstract

Currently, consumers appear to have diversified characteristics with regard to food tastes and consumption habits. The globalization of markets and the migration phenomenon contributed to the modification of food preferences of consumers who gradually introduce into their eating habits foods and recipes typical of the tradition of foreign countries. In this scenario, also in Italy, it is going to increase the use of “foreign products” with the consequent fusion of traditional cuisine techniques and recipes with ingredients that are typical of foreign countries and cultures. Foods and ingredients originally consumed in Asian or South American countries are increasingly consumed by Italian people, who have notoriously a strong “food identity,” who generally follow the Mediterranean Diet, and who consume typical products of the country. More particularly, the use of “new” or “novel” spices compared to those traditionally used in the preparation of Italian food has grown in the last 10 years. This study is an exploratory survey on the consumption of spices in Sicily (Southern Italy), which is an Italian Region with a high level of immigrated people and a good level of social integration and progressive inclusion. The objective of this study is to know whether and how Sicilian consumers’ consumption preferences, use, and purchasing behaviors with regard to spices changed in the last few years. The results highlight an increasing use of novel spices for the preparation of the traditional recipes and a good appreciation by consumers. These results are interesting because they provide information about spices’ market development and food product marketing and internationalization. The survey gives interesting inputs for reflections about the relationship between food contaminations and social integration and insight into consumers’ preferences in Italy.

## Introduction

Over the last 20 years, due to globalization, consumers’ changes in lifestyle and habits involved changes in food preferences and consumption. The actual consumers’ lifestyles lead to prefer “quality foods” in terms of healthier, lighter, and safer food [1]. *Food culture* in Italy means to pay more attention to maintaining ancient traditions in the production of food and in the recovery of ancient traditional recipes [2]. Literature shows that consumers’ preferences are largely diversified and so are their food consumption choices. On the one hand, consumers are more concerned towards consumption of healthy and nutritious foods, which are processed using production techniques well matched with environmental protection and respect for social needs [3]. On the other hand, the globalization of markets and the migration phenomenon contributed to the modification of consumers’ food preferences. The Italian consumer, who has notoriously a strong “food identity,” who generally follows the “Mediterranean Diet” nutritional model, and who consumes typical products of her/his country, introduced progressively into her/his daily diet and food habits products and recipes generally belonging to the food culture of other populations [4]. At this time, in Italy, besides the Mediterranean Diet, which is a nutritional model inspired by the dietary models widespread in some countries of the Mediterranean basin (recognized by UNESCO as a protected asset and included in the list of oral and intangible heritage of humanity in 2010) [5], ethnic contaminations are going to be well integrated into traditional flavors and food preparations [6, 7]. A slow and natural process of inclusion and integration of eating habits is going to be realized. Food consumption is the bearer of social and cultural values that, at the same time, involve the nutritional, hedonistic, social, and symbolic spheres of modern consumers.

## Literature review

The origins of spices are truly ancient and going back in time, their history lead to the Far East where spices were considered a rare and precious commodity. For millennia, these colorful powders with “prodigious” properties were used as medicaments, cosmetics, or food condiments. Spices were already well known since the time of ancient Sumerians, Egyptians, Phoenicians, Persians, and Chinese populations, who used them for the treatment of seasonal diseases [8, 9]. These populations offered spices as gifts to gods and used them for the preparation of perfumes [8, 9]. The ancient Egyptians used them to embalm the bodies of the dead [8, 9]. The Phoenicians started the first commercial routes of spices in the Mediterranean Sea. With the Greeks, who transported them by sea from the East to the Mediterranean, they became a very prosperous and profitable market. The Romans were the first to use spices in the kitchen: black pepper, cinnamon, coriander, anise, mustard, and cloves were the preferred ingredients for flavoring the dishes [8, 9]. Always the Romans discovered the use of spices also as preservatives for food [8, 9]. Ultimately, the history of the spices’ rush was, first of all, a story of population and cultures which, enchanted by the charm of these colorful and miraculous powders, met and collided to conquer this a precious asset, such as spices, that were considered bearers of economic wealth [8, 9]. In most of the scientific and commercial literature, there are no clear distinctions between aromatic herbs and spices; in fact, some of them fall into the same category. As we said, spices are native to the East of the world and are vegetables derived from different parts of aromatic plants (e.g. seeds, roots, leaves, fruits, bark, or berries) depending on the type of spice obtained, for example, bark for cinnamon, roots for ginger, floral buds for cloves, and saffron, seeds for sesame and mustard, berries for black pepper, fruits for paprika [10]. Unlike spices, most aromatic herbs are native to the Mediterranean basin area and are consumed as fresh, e.g., basil, parsley, rosemary, sage, and thyme. The U.S. Food and Drug Administration (FDA) does not provide identity standards for spices, but it provides a guide of terminology that can be used for product labeling [11]. The FDA defines spice as an aromatic plant substance, in whole form, broken, or ground, which has the main function to be used to season foods; no essential oil or other flavoring element is extracted from the spice. The United States National Arboretum offers an alternative definition, describing spices as dried aromas and aromatic herbs as fresh or dried leaves from plants that can be used to flavor foods [12]. The Codex Alimentarius Commission, also known as CAC, is the central part of the joint FAO-WHO International Food Standards Program, and it was established by the FAO and the WHO with the aim to protect consumer health and promote fair practices in the food trade. The Codex Alimentarius Commission is trying to harmonize spices standard around the world [13]. This Commission adopted the Codex Alimentarius, or “Food Code” which is a collection of standards, guidelines, and codes of practice. Codex standards ensure that food is safe and can be traded [13]. With regard to spices, the Codex Committee elaborated worldwide standards for spices and culinary herbs in their dried and dehydrated states in whole, ground, and cracked or crushed forms and operates to avoid duplications in the standards development process [13].

Thanks to market globalization, today, most spices are easily available in any country worldwide. The global spice market is growing at an annual growth rate of about 5% in terms of value and it is estimated to exceed $10 billion by 2020. In addition, the global market for spices and herbs will grow at a considerable speed during the forecast period between 2020 and 2026 [14]. Over the years, the global spice trade showed a significant and differentiated product development. With production industrialization, the industry of spices became more consolidated. Moreover, thanks to the development of Information and Communication Technologies (ICT) and Business To Business (BToB) activities, the demand and supply of spices increased rapidly within importing and exporting countries; this contributed to enhance the whole trade of spices and make the spice market more and more competitive. Nevertheless, according to literature [15] there are around 40-50 spices of global economic and culinary importance and many lesser known spices used in traditional cooking that have little or no international trade value. Herbs and spices help to vary dishes and to emphasize the national particularities of ethnic cuisines (e.g., turmeric in Indian cuisine; basil, garlic, and oregano in Italian and Greek cuisines; wasabi in Japanese cuisine; and paprika powder in Hungarian cuisine). At the same time, spices enrich food with important nutrients for the body, and in some cases, they are used to lengthen the time storage of food [16]. Additionally, spices are very valuable for the cosmetic industry and are used for medical solutions or as dietary supplements. In fact, spices are used for their high antioxidant activity [17] in different forms, like essential oils, aqueous or methanolic extracts, resins, whole, and oleoresins in food. In fact, spices have a large number of bioactive compounds such as flavonoids, phenolic compounds, sulfur-based compounds, tannins, alkaloids, and vitamins [18, 19]. Furthermore, today some spices are classified as *superfoods*, although there is not a standard definition of these special foods, and generally, they are considered a sub-category of “functional foods.” Principally, superfoods have a high nutritional content with benefic properties to which the concept of quality is linked, which is absent in functional synthetic foods [20]. Superfoods have a vegetable origin, and thanks to the particular intrinsic characteristics, they give the human body a large amount of vitamins, minerals, essential and non-essential amino acids, fatty acids, and useful phytochemicals [21]. Superfoods are fruit, spices, seeds, and berries; the list of these foods is constantly evolving because the science constantly discovers new antioxidants and nutrients within some foods [21, 22]. Superfood consumption is an expanding phenomenon in Italy due, for the most part, to the introduction into our market of new raw materials, originating from distant cultures, discovered or reintroduced in our daily consumption [23, 24]. Superfoods do not undergo technological processes, and therefore, they are natural foods, opposite to foods produced using modern technology [16, 25].

**Fig. 1:**
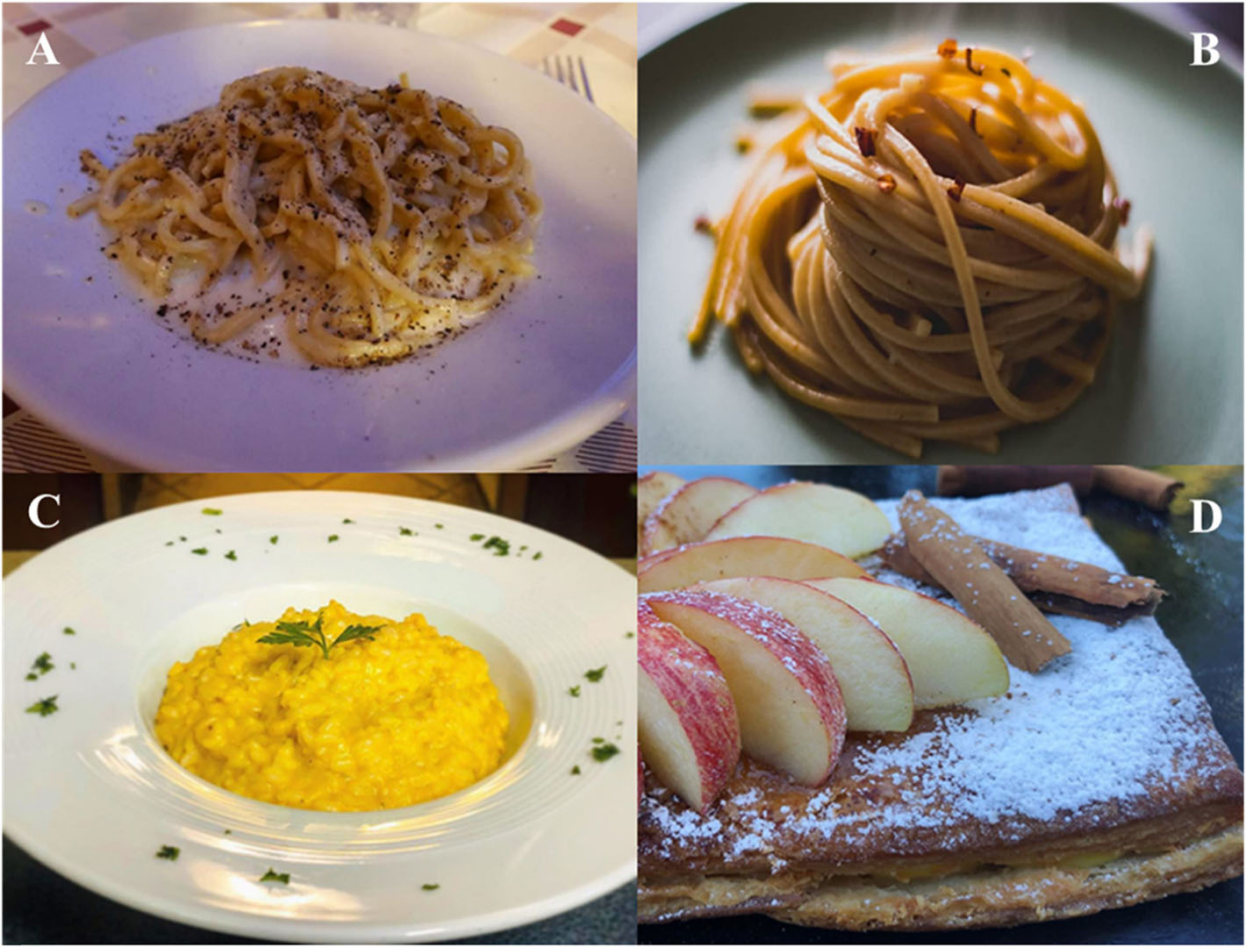
Typical dishes of traditional Italian cuisine where spices are used

Although consumption of spices is generally a prominent part of the traditional cuisine of Asian countries (China, Thailand), Africa (Northern Africa), India, and Mexico, in recent years, the trend of consumption increased also in European countries, where the gastronomic and food traditions do not include a large use of spices in food preparation. The slow and constant introduction and integration of spices typical of non-EU countries in Europe [15, 26] could be explained by the ongoing change in consumers’ food preferences and consumption habits, which have turned traditional tastes leading them to ethnic and spicy flavors [27]. The spices most consumed in Italy are those used traditionally in the Italian recipes, such as pepper, chili pepper, and nutmeg mainly for Pasta (Fig. 1), saffron for the first course of rice (Fig. 1), sesame for bread and sausages, and cinnamon for sweats (Fig. 1).

Picture **A** shows one of the most famous Italian traditional first course, which is Pasta with Italian typical cheese named *Caciocavallo* (this cheese is aged *pasta filata* cheese typical of southern Italy, produced with cow’s milk with the addition of only rennet, milk enzymes, and salt; it is preserved hanging on horseback of a beam for drying and is, consequently, shaped with two round bodies joined by a bottleneck in the point of support on the beam) and pepper; this course is named “*Pasta Cacio e Pepe*.” Picture **B** shows another famous and typical first course of Italian cuisine made of pasta with garlic and chili pepper, named “*Pasta Aglio, Olio e Peperoncino*”; this plate is made also with Italian olive oil. Picture **C** shows a first course which is typical of Italy but originating from Milano (Lombardy Region, North Italy), which is made of rice with cheese and saffron, named “*Risotto alla Milanese*.” Picture **D** shows a sweet, named “*Pesca con zucchero a velo e cannella*,” made of sweet puff pastry with flesh white fresh peach, powdered sugar, and cinnamon.

Nevertheless, in the last decade, also in Italy, the consumption spices originally used in foreign countries, such as turmeric and ginger, has increased thanks to the growing consumers’ interest in ethnic food [28]. Food culture evolves through a social and ethnic mix, and it is influenced by new lifestyles and consumption models able to modify, progressively, the traditional eating behaviors [1–29]. Although in Italy traditional food consumption patterns persist, the new consumers’ lifestyles have fostered the introduction of “novel” foods and “novel” recipes into the traditional diet and food preparation [30]. The factors that influenced this process are a frequent consumption of lunch and dinner outside the home, the need to prepare food in a short time at home, the consumption at home of products purchased from takeaway restaurants, which in Italy, apart from pizza, are “American” burgers, Chinese food, Mexican food, sushi, and other foods taken from ethnic restaurants. These preferences highlight the profile of a consumer who is increasingly interested in learning new ways of consuming and preparing food and experimenting with new flavors through diversified sensory experiences, thus representing a new model of consumption [31]. Moreover, although today the use of spices is increasingly frequent, it is clear that in Italy the use of some of them is more frequent or preferred with respect to others that, contrarily, are of fundamental importance for the preparation of food in other countries, such as curry in China or India, chili in Africa, ginger in Japan, etc. [32, 33].

In Italy, the island of Sicily (Southern Italy) has always been the crossroads of different peoples. Sicily, due to its geographical position, is a region with a high level of inclusion and integration among different food cultures [34]. Since prehistoric times, in fact, Sicily has known a flourishing life, thanks to populations from the Middle East and northern Europe, who grafted their exotic and heterogeneous cultures, and that characterized the period from 2200 to 1900 BC. Subsequently, Sicily was a Phoenician colony, where this population placed emporia (i.e., places used for unloading, storing, and selling goods) and made this land the heart of the prosperous trade in the Mediterranean basin [34]. Later, starting from the second half of the eighth century BC, Sicily was one of the Greek colonies, and then, different peoples and cultures have followed one another on this island, such as the Romans, the Vandals, the Ostrogoths, the Byzantines, and the Muslims, which deeply marked the food culture of Sicilian people. Over time, French (the Orleans dynasty), Swedish (the Altavilla dynasty), and Spanish dominations followed one another [33]. Fusion among populations and food contaminations in Sicily have been favored by the fact that Palermo, the city that today is the capital of the region, has always been a city with a pleasant climate and a welcoming and safe natural harbor, so much so that it was originally called *Zyz* by the Phoenicians, meaning the flower, and its current name comes from the Greek word Panormos (παν-όρμος) that means “all-port,” due to the presence of the two rivers Kemonia and Papireto that created a huge natural landing place, which became Panormus with the Romans [34]. Today, Palermo also preserves architectural treasures that demonstrate the presence and long stay of these peoples; one among many is the famous Arab-Norman route that today has become one of the sites on the list of the UNESCO World Heritage Sites [5]. At the end of 2019, there were 37,092 foreign citizens/residents living in Palermo [35]. Considering the total number of residents, the number of immigrants is rather low, standing at 3.9%. The most representative groups come from the Indian subcontinent, led by Bengalis and Singhalese^1^. Moreover, in Palermo, the following Consulates represent the internationality of the city and the island: Albania, Austria, Bangladesh, Belgium, Bolivia, Brazil, Bulgaria, Burkina Faso, Cape Verde, Czech Republic, Chile, Cyprus, South Korea, Denmark, Estonia, Finland, France, Germany, Ghana, Greece, Libya, Lithuania, Luxembourg, Macedonia, Malta, Morocco, Mexico, Netherlands, Norway, Pakistan, Poland, Portugal, Monaco, UK, Russian Federation, Senegal, Slovenia, Spain, USA, South Africa, Sweden, Tunisia, Zambia. Today Palermo has a large Civil Port, which is one of the largest ports for passenger traffic and size of the Mediterranean and as many as 8 tourist ports; and in Sicily, there are also 8 major ports of significant size for freight and passengers and 3 international airports in Palermo, Catania, Comiso (RG), and Trapani. There are several studies in the literature on the culinary use of spices and on their beneficial properties and alternative uses in the food sector; nevertheless, there is few literature on marketing studies on the consumption of spices in Italy. This study is an exploratory survey on the spice consumption in Sicily. Today, in Sicily, the typical Italian cuisine represent the regular food consumed, even if people often consume dishes belonging to local culinary tradition prepared using “typical” recipes. The aim of this study is to know Sicilian consumers’ preferences, consumption and purchasing behaviors, and dietary habits with regard to spices and the level of knowledge of consumers about spices in order to know the potential of development of commercialization for these products.

## Methodology

### Data collection and sample

The survey was carried out from July 2019 to December 2019 and it was directed specifically to Sicilian consumers; a sample size of n ≥ 800 was established for this study, considering the population of residents in 2019 *N* = 4.999.891, confidence level 95%, and sampling error ≤ 3%.

A proper questionnaire was prepared by the research group. The questionnaire, prepared using Google Form was spread through the internet, by email, and some by social media. The snowball sampling was applied. A total of 962 individuals (statistical units or units) completed the questionnaire 47 questionnaires were excluded because after quality control of the collected data; the units whose answers contained errors, contradictions, blank fields, or those repeated (questionnaires sent two times or more at same registered hour/time with same answers — “replicates”) were eliminated. This procedure resulted in an effective database/sample of 915 respondents/questionnaires.

Subsequently, using the simple random extraction method from the database of 915 units, a sample size equal to *n* = 877 was set up and it was balanced with respect to gender, age, education, and income (see Table 1; Pearson’s χ^2^ test was used for balancing). The application of the simple random method for the final extraction allowed us to make an inference (in order to try to draw a conclusion on the starting population based on information obtained from a sample). The applied balancing criteria increased the accuracy of the estimates.

**Table 1:** Description of spices observed: name, country of origin, use in traditional Italian cuisine, beneficial properties, and picture^a^

Spice name	Country of origin	Use in the traditonal Italian cuisine	Beneficial properties	Picture
Acai berry	South American rainforest	Berries are used for the preparation of smoothies, fruit salads, to enrich yogurt	antioxidant and energizing properties	
Anis	Far East	used to prepare liqueurs and digestives with an aromatic scent such as Anisetta and Sambuca which are used to flavor various desserts, including the famous biscuits known as anicetti (typical of Marche Region cuisine) used to flavor bread and cakes used to flavor cheeses and vegetables.	digestive and stimulating properties excellent cough remedy excellent aphrodisiac properties	
Black garlic	Korea	used cooked in the spaghetti pasta of the typical Italian cuisine with oil and chili or in soups	considered a superfood useful for reducing cholesterol antioxidant and diuretic properties helps prevent cardiovascular disease used for the treatment of psoriasis	
Cardamon	India and Malaysia	used to prepare meat main courses (e.g. roasts) and goes perfectly with citrus fruits and chocolate in desserts.	useful for helping digestion and the intestine aphrodisiac properties	
Chia	Central American countries	main use in the preparation of sweets	considered a superfood anti-colitis, anti-cholesterol and slimming properties preventive action against diabetes and osteoporosis anti-inflammatory action rebalancing of hormones for the pre-menopausal woman	
**Chili pepper**	American continent	used to flavor dishes giving spiciness and color in Mediterranean cuisine, it is used to flavor sauces and condiments for first courses but also meats and cheeses	natural remedy to stimulate blood circulation balancer of level of cholesterol in the blood and helps to activate the metabolism	
**Cinnamon**	Sri Lanka	very particular flavor, it is spicy and sweet at the same time mainly used for making desserts: cakes, biscuits, fruit, candies and creams it can give a particular flavor to ice cream, fruit salad and *ricotta*	excellent natural remedy for colds relaxes the nervous system	
Cloves	Asia	used for flavoring meats and to prepare broths with a strong flavor excellent with vegetables widely used for the preparation of desserts, especially based on apples	facilitate digestion possess anti-inflammatory properties help reduce gastric acidity help keep sugar and cholesterol levels low help relieve toothache natural anti-aging	
Coriander	Mediterranean countries	used to flavor soups, meat and fish and cured meats also used for the preparation of biscuits or the typical Sienese cake *Panpepato*	therapeutic properties antibacterial anti-fermentation action in the intestine able to chelate heavy metals in the body	
Cumin	Asia minor and Syria	multiple uses in the kitchen, thanks to their intense and slightly spicy flavor widely used in Indian cuisine little use in the Italian cuisine mainly used to flavor bread and cheeses	with several healing properties excellent defenses for the immune system and anemia digestive and purifying properties great for reducing stress powerful anti-inflammatory spice	
Curry	India	spicy flavor blend used in addition to various types of food to enhance taste it can be used for both savory and sweet recipes	anti-inflammatory, antioxidant and antibacterial properties helps to maintain good functions of the digestive system useful in the treatment of joint diseases and muscular problems	
Ginger	Far East	it can be used grated, on first courses or to prepare salads, but it can be used to prepare drinks and infusions	digestive and stimulating properties excellent anti-inflammatory	
Goji berry	Asian highlands of Tibet, China, and Mongolia	used mixed with other fruits, in yoghurt, fruit salads, and for the preparation of smoothies. also can be used to prepare slimming herbal teas	considered as a super food good antioxidant properties help immune defence revitalizing effect useful for low content calories diets	
**Mustard**	Mediterranean basin area (Southern Europe and Northern Africa)	used to make mustard to enhance the taste of meat or fish main courses or to enrich salads	help stimulate blood and lymphatic circulation; they help fight joint and muscle pains	
**Nutmeg**	Indonesia	used as an ingredient for the preparation of sweets; it is also often added to fillings for *ravioli, tortellini*, and *lasagne*	therapeutic, antiseptic, stimulating and aphrodisiac properties if it is ingested in massive quantities it can cause hallucinations	
**Pepper**	India	broths, salami and sausages are enriched with whole seeds while the ground seeds are used for flavor meat and fish used for the preparation of the traditional Roman pasta “spaghetti with cheese and pepper” also used to season soups and risottos excellent for giving more flavor to cheeses and vegetables such as fennel, carrots and peas	helps to promote digestion and stimulates metabolism excellent natural antidepressant	
Poppy seed	Mediterranean basin area	mainly used to give flavor to baked goods such as bread, breadsticks and some typical traditional products also used as a dressing for salads, soups or they can be eaten with yogurt or in milk for breakfast	help keep cholesterol under danger level indicated for bone health promote metabolism excellent antioxidant properties help against anxiety and stress	
**Saffron**	Asia minor and Greece	used in the preparation of main courses particularly used to flavor first courses (it is used in the *Risotto alla Milanese*) another use is in the recipe of *Sicilian Arancine*	excellent antioxidant, anti-stress, digestive and energizing also used to control body weight	
**Sesame**	Tropical countries	excellent for flavoring bread, pasta and risotto used for preparing breading for chicken, meat or vegetables used to flavor pasta rice, and main courses of meat and fish	high content of omega 3 valuable properties for the prevention of heart disease, osteoporosis calming and refreshing action	
Turmeric	India	used to flavor vegetables, to flavor tofu or in the preparation of crackers and breadsticks	excellent antioxidant healing properties digestive and anticancer properties	
**Vanilla**	Mexico	excellent for making cakes or biscuits; it can be added to smoothies, cold beer, cocktails and hot chocolate	aphrodisiac, antidepressant, anticancer, antioxidant properties	
Wasabi	Japan	it was introduced in the Italian cuisine in recent years used in the form of sauce or accompany sushi and sashimi	anticancer action used to improve diuresis and counteracts water retention excellent antioxidant	

^a^This table shows the spices used in this study, describing for each spice its benefic properties and the use in Italian cuisine. Particularly, the spices with names in bold are used in the Italian traditional recipes

### Measurements

The questionnaire was structured containing closed and open-ended, alternating, or multiple-choice questions with filter and barrier questions. Specifically, the questionnaire consisted of two sections, the first one containing questions related to the demographic characteristics of the sample and questions concerning the consumption of spices. The second part contained questions on the following topics: motivations/preferences; type of use/consumption behavior, as well as purchasing habits. Motivations and preferences were measured using a 5-point importance/preference scale [36] with this caption to facilitate understanding of the scale: *1 = not at all, 2 = slightly important/preferred, 3 = moderately important/preferred, 4 = discreetly to highly important/preferred, 5 = extremely important/preferred*. The average of responses to these five items with the calculated sample size of 877 had good construct reliability (Cronbach’s alpha = 0.85). The so structured questionnaire was tested by a panel of qualified subjects, i.e., people with particular knowledge and/or competence on the treated subject, in order to check consistency and coherence. Subsequently, a pre-test of the questionnaire was carried out with the method of self-filling to a small sample of individuals extracted with a random method within the university campus, with the aim of verifying the clarity and unambiguity of the questions and the understandability of the language used as well as the suitability of the type of response of each question in order to simplify and speed up the process of response by the interviewed persons. Statistical analysis was performed with IBM SPSS Statistics version 21 (SPSS Inc Chicago IL, USA).

For this study, 22 types of spices have been selected, which are those available in the Italian market, specifically açaí berry, anise, black garlic, cardamom, chia, chili pepper, cinnamon, cloves, coriander, cumin, curry, ginger, goji berry, mustard, nutmeg, pepper, poppy seeds, saffron, sesame, turmeric, vanilla, and wasabi (Table 1).

## Results and discussions

Socio-demographic characteristics of the sample are shown in Table 2.

**Table 2:** Socio-demographic characteristics of the sample, distribution in percentages

Variables	Pct. (%)
*Gender*	
Male	48.0
Female	52.0
*Age*	
18–24	13.7
25–34	21.6
35–44	24.0
45–54	18.8
55–64	16.9
> 64	4.9
*Education*	
Primary school	2.9
Lower secondary school	5.5
Higher secondary school diploma	20.8
University diploma/degree	61.5
Master degree or above	9.3
*Income*^b^	
Low	13.0
Intermediate	78.9
High	8.1

^b^Ranges of income. Low: under 20,000 Euros; medium: between 20,000 and 60,000 Euros; high: over 60,000 Euros. This table shows the socio-demographic characteristics of the sample selected; educational level and income were used as parameters to select the sample

Most of the sample, 62% of respondents, do grocery shopping by themselves; of these, over half do food shopping 1–2 times per week (data not shown). Ninety-six percent of the sample declared to consume spices regularly and a minority of respondents said not to use them because they do not like spices (data not shown). Among those who regularly consume spices, about 94% have always used them for food preparation. Since some of the spices examined have always been habitually used in the preparation of dishes, because they belong to the traditional Italian cuisine, results show that these spices are the most frequently used both for preparing seasonings for pasta or sides for second dishes or sweets. These spices are pepper (93.8%), chili pepper (82.2%), saffron (74.2%), cinnamon (69%), nutmeg (68.8%), vanilla (65%), sesame (59.4%), cloves (42.5%), and anise (33.6%).

Some spices have been part of regular consumption for more than one year in the preparation of common Italian dishes, such as curry (49.7%), ginger (49%), and turmeric (47.4%), along with cumin (26.1%), wasabi (25%), poppy seeds (22.2), and chia (16.3%), which are the so-called new/novel spices (Table 3). Finally, spices such as goji berries (17.7%), black garlic (16.8%), and açaí berries (79.9%), which have good healthy qualities, are little consumed because they are not always easy to find on the market, because they have higher prices and, in any case, they are less known by most consumers (Table 3).

**Table 3:** Frequency of spice consumption over time^c^

Spices	Always consumed	Consumed for more than 1 year	Consumed for less than 1 year	Not consumed
Açaí berry	3.4%	3.4%	13.2%	79.9%
**Anise**	33.6%	9.2%	12.4%	44.7%
Black garlic	9.4%	6.9%	16.8%	66.8%
Cardamom	8.9%	18.7%	11.8%	60.6%
Chia	4.1%	16.3%	16.3%	63.3%
**Chili pepper**	85.2%	9.3%	2.4%	3.0%
**Cinnamon**	69.0%	14.5%	5.9%	10.6%
Cloves	42.5%	19.7%	7.3%	30.5%
Coriander	19.8%	23.0%	12.0%	45.2%
Cumin	19.8%	26.1%	11.3%	42.8%
Curry	33.9%	49.7%	6.6%	9.8%
Ginger	26.9%	49.0%	10.9%	13.3%
Goji berry	4.4%	22.7%	17.7%	55.2%
**Mustard**	39.1%	18.3%	10.2%	32.3%
**Nutmeg**	68.8%	17.9%	3.9%	9.4%
**Pepper**	93.8%	2.8%	2.8%	0.6%
Poppy seeds	21.7%	22.2%	10.4%	45.8%
**Saffron**	74.2%	12.9%	4.5%	8.4%
**Sesame**	59.4%	16.6%	6.6%	17.3%
Turmeric	20.0%	47.4%	13.0%	19.6%
**Vanilla**	65.0%	11.7%	5.6%	17.7%
Wasabi	9.2%	25.0%	13.3%	52.6%

^c^This table shows all the spices selected for the study. Spices indicated with bold letters are those consumed for longer or always in the Italian traditional recipes over time

With regard to frequency of consumption, among those respondents that declare to consume pepper, about 39.3% of respondents consume it every day, and 40.2% with a frequency of 1–3 times a week (Table 4). 34.1% of respondents (Table 4) consume chili pepper daily and 22.6% sesame; however, these spices are the most commonly used for local cuisine.

**Table 4:** Frequency of consumption (%)^d^

	Everyday	Once to 3 times a week	Once to 4 times a month	Rarely	Never
Açaí berry	0.0%	1.6%	11.8%	5.3%	81.3%
**Anise**	1.4%	4.5%	21.8%	27.7%	44.5%
Black garlic	2.4%	5.4%	14.1%	8.8%	69.3%
Cardamom	0.9%	6.5%	19.0%	16.7%	56.9%
Chia	4.8%	5.8%	13.5%	11.5%	64.4%
**Chili pepper**	34.1%	39.3%	18.3%	4.9%	3.4%
**Cinnamon**	3.0%	16.6%	41.9%	29.2%	9.3%
**Cloves**	1.6%	9.6%	28.8%	30.4%	29.6%
Coriander	2.2%	10.2%	23.6%	15.1%	48.9%
Cumin	2.2%	12.9%	21.0%	18.8%	45.1%
Curry	5.1%	28.6%	34.8%	23.9%	7.6%
Ginger	11.2%	27.8%	33.6%	15.6%	11.9%
Goji berry	1.9%	5.6%	19.1%	16.3%	57.2%
**Mustard**	2.6%	14.7%	30.3%	19.5%	32.9%
**Nutmeg**	4.7%	30.3%	37.4%	18.5%	9.1%
**Pepper**	39.3%	40.2%	15.5%	3.3%	1.8%
Poppy seeds	3.6%	11.3%	23.5%	15.8%	45.7%
**Saffron**	2.9%	26.6%	42.9%	20.1%	7.5%
**Sesame**	22.6%	20.3%	23.0%	15.7%	18.4%
Turmeric	9.3%	23.4%	29.4%	18.2%	19.7%
**Vanilla**	4.6%	20.4%	36.2%	22.7%	16.2%
Wasabi	1.5%	7.5%	20.4%	17.9%	52.7%

^d^This table shows all the spices selected for the study. Percentages show the frequency of consumption and use by Italians during a week in the preparation of dishes. Spices indicated with bold letters are those belonging to traditional Italian cuisine

Moreover, it is interesting to note (Table 4) that some of the novel spices are consumed with a frequency of at least 1 to 4 times a month, for example, saffron (42.9), nutmeg (37.4%), vanilla (36.2%), curry (34.8%), mustard (30.3%), and turmeric (29.4%).

Ginger was recently introduced in the feeding, and it is consumed every day by about 11.2% of respondents, from 1 to 3 times a week for 27.8% of respondents and from 1 to 4 times a month by 33.6% of the interviewed consumers. This can be explained by analyzing the results of the second part of this research. In fact (Table 5) it can be consumed in different occasions thanks to its hot, balsamic, and spicy taste. Ginger is used to prepare sweets, drinks, salads, or second dishes in general. Furthermore, its properties are well known by consumers that use this spice also for its beneficial effects, for its anti-inflammatory power or to strengthen immune defenses, or also as an infusion to facilitate digestion (Table 5). As far as the second part of the survey is concerned (i.e., the one aimed at knowing motivations/preferences of consumers, type of use, consumption behavior, as well as purchasing habits), it is clear from the results that the highest preference was expressed for the item “enhance the taste of food” (50% extremely important/preferred and 13% discreetly to highly important/preferred), particularly with regard to the second course (41%), followed by “preparation of unique course,” “preparation of first course,” “preparation of herbal teas,” and “preparation of desserts” (Table 5).

**Table 5:** Motivations, preferences, types of use/consumption habits, and purchasing habits (% of ratings)

Motivations–preferences–types of use/consumption–purchasing habits	Extremely important/preferred	Discreetly to highly important/preferred	Moderately important/preferred	Slightly important/preferred	Not at all
Enhance the taste of food	50	13	23	5	2
Boost the immune system	22	15	28	15	25
For its antioxidant power	21	12	33	16	18
Reduce the use of salt	18	11	30	27	14
Aid digestion	14	13	25	18	30
Preparation of second course	41	18	31	8	2
Preparation of unique course	35	20	30	10	5
Preparation of first course	33	20	37	6	3
Preparation of herbal teas	15	15	30	10	30
Preparation of desserts	10	10	35	25	20
Bulked spices allow to choose the perfect quantity to buy	50	10	5	5	30
Packaged spices are easier to use/store at home	40	15	15	5	25
Bulked spices have higher quality	36	27	23	15	5
Packaged spices have information in the label	25	15	20	20	20
Bulked spices are cheaper	15	10	10	10	55

It is interesting to note that results highlighted a good interest in the beneficial properties of spices (Table 5). In fact, respondents declared to use spices because they are useful for strengthening the immune system (22% of extremely important/preferred, 15% of discreetly to highly important/preferred, 28% of moderately important/preferred), and for their antioxidant power (21% extremely important/preferred, 12% discreetly to highly important/preferred, 33% moderately important/preferred). This is in line with results of other studies on consumers’ preferences for fruits and vegetables [37–39] and it is linked to the new consumers’ habits of food preparation. These results highlight an Italian consumer who is interested in healthier diets, and who uses ingredients that are easier to digest, less harmful to the body, or even beneficial in the long term. Particularly, results show that according to respondents, reducing salt consumption in the daily diet is extremely important 18%, highly important 11%, and moderately important 30%. Moreover, the item “aid digestion” received 14% of extremely important/preferred, 13% of discreetly to highly important/preferred, and 25% of moderately important/preferred. Consumers expressed a good preference also for the preparation of herbal teas (15% extremely important, 15% highly important, and 30% moderately important) (Table 5).

Concerning the type of product purchased (Table 5), results revealed that the interviewees showed a particular interest in bulk spices because it is possible to “choose the perfect quantity to buy” (50% extremely important/preferred, 10% discreetly to highly important/preferred and 5% moderately important/preferred) and because consumers think that “Bulked spices have higher quality” (36% 27% 23%). Maybe this is related also to a sensorial appreciation; in fact, consumers that buy bulk spices at markets, have the possibility to test sensory attributes of spices and use their sensory qualities as elements for choosing [40]. Contrarily, consumers think that the bulked product is more expensive; in fact, 55% of respondents declared that it is not cheaper than the packaged one. However, it is interesting to observe that consumers expressed good preferences also for packaged spices, because they are easier to store and handle when dosing, and because in the labels, it is possible to find useful information, e.g., the intrinsic characteristics of the product and the different ways of usage (40% of extremely preferred and 15% of highly preferred). This information is in line with marketing studies [41–43] and appears to be important for consumers that do not consume this product habitually. At the same time, this information can be important for producers and distributors because consumers’ insight of needs and desires helps for a larger diffusion and commercialization of this product.

As far as the importance of different ways to know and learn about the “novel” spices (Table 6) is concerned, respondents said that ethnic restaurants appear extremely important (25%) and highly important (27%). Travel is another fundamental vehicle of knowledge (20% extremely important, 20% discreetly, and 20% moderately important); this is understandable given the globalization and the ease of traveling around the world. In fact, thanks to the ease of traveling for work or tourism in the last 20 years, most Italians have been able to experience and appreciate foreign spices and cuisines during travels and test the original dishes in each country of origin. Unfortunately, in the last months of 2020, there was no longer the possibility for people to travel from one continent to another in the world due to the COVID-19 pandemic; nevertheless, this does not cancel out the current “potential” ease of long-distance travels compared to 30 or 20 years ago. Following with the preferences, 40% of respondents said that TV programs are moderately important, 35% discreetly to highly important/preferred, and 5% think they are extremely important (Table 6). The “word of mouth” is also moderately important (Table 6), since it is well known that consumers trust the advice of those who have already tried the product. Finally, consumers think that the Internet is less important than the other means of information through which they knew novel spices (Table 6). Maybe the consumer uses the Internet mostly to have information about how to use spices for food preparation or to have information about spices’ healthy properties and this is not the consumer’s first source of information/knowledge.

**Table 6:** How consumers knew about the “novel” spices (% of ratings)

Vehicles of knowledge	Extremely important	Discreetly to highly important/preferred	Moderately important	Slightly important	Not at all
Ethnic restaurants	25	27	20	15	13
Travel	20	20	25	15	20
TV programs	5	35	40	10	10
Word of mouth	20	20	30	20	10
Internet	10	23	28	20	19

Alongside these numbers, however, is the progressive high introduction of ethnic restaurants in the Italian cities, particularly in those where the presence of foreign people coming from countries outside Europe is very high, like the case of Italy and specifically Sicily (Southern Italy )[44].

## Discussion

This study provides first information about the integration of the foreign gastronomic culture with the Italian food traditions in Sicily. This appears a very interesting result that highlights the effects of globalization, and at the same time, those of large migration flows that have as their destination Italy and Sicily as the first place of arrival very often.

Results revealed interesting information about consumer behavior and preferences with regard to spices. In fact, the study highlighted that consumers decide to buy and consume spices at home intentionally—the sample declared to be responsible for the purchase of groceries—and they are aware of the use they are going to do in their recipes and food preparation. The results have shown that the most consumed spices are those that are part of traditional Italian cuisine. However, results also revealed that the consumption of the so-called novel spices in Italy has increased in recent years favored by the intersection of social and cultural factors that have developed simultaneously.

Consumers’ interest is often stimulated by direct contact with product; in fact, it was observed that spices are also preferred to be purchased in bulk and at a market, maybe also because of greater savings; the combination of colors, smells, and flavors involve consumers to a complete sensory experience [1, 40, 45]. Nevertheless, it is also possible that TV broadcasts and diffusion of ethnic restaurants (besides traditional Italian restaurants) also influenced the desire to use spices and handle bulk spices. Specifically, this trend, in agreement with other authors, could be explained by the fact that the diffusion of TV programs wherein famous chefs teach how to cook. During the shows, chefs tell the stories of the recipes of the prepared foods or explain the content of the raw materials used in the preparation, also touching with hands the raw material used in the preparation of the dishes. Therefore, they attract viewers and lead them to introduce new flavors and aromas in their traditional recipes for experiencing sensorial novelties. These results reflect other studies related to the consumption of ethnic foods in Western countries and the role that mass media play in the diffusion and consumption of ethnic foods [46].

Another important element that plays a key role in the spread of the use of new/novel spices is the diffusion of ethnic restaurants and the change in the consumer’s lifestyles and consumption habits. In fact, consumers are increasingly inclined to consume meals away from home and therefore want to experiment with different restaurants, perhaps themed or where it is possible to consume novel foods, such as Mexican, Chinese, Japanese restaurants, etc. Moreover, changes in consumers’ lifestyles induced people to consume take-away meals at home where take-away food is typical of different food cultures.

The results furthermore highlighted that the fusion of different foods and elements is appreciated by consumers that are always open to new food experimentations; in this case, typical foods and tastes of different food cultures are mixed with Italian foods, generating the so-called fusion food or food mixology [47].

Moreover, according to previous literature, also for spices results revealed that the link between nutrition, health, and wellness strongly influenced consumers’ eating choices [48, 49]. This is also linked to the preference for unconventional foods, such as superfoods, that promise beneficial effects as anti-age and as supplements to support the good metabolism of the body for the individual wellness and may be related to the idea that bulk spices can be of higher quality than packaged ones [30]. Like in other studies, also in this case results highlighted that a large part of consumers is not informed and aware of healthy properties and nutraceuticals of foods. More particularly, with regard to this study, results revealed consumers are not fully informed about beneficial effects that spices can have if used constantly in the human diet because of their nutritional intake as an adjuvant for the prevention of certain diseases or to maintain the right amount of nutraceutical elements in the daily diet. However, in agreement with other studies on other foods [50, 51], it appears also in this study that when consumers are informed about the healthy properties of some foods, they are willing to buy them with awareness and sometimes willing to pay a higher price for them. Therefore, information on the characteristics and health benefits of spices appears to be important.

Furthermore, according to other recent studies on ethnocentrism and product knowledge [52], also in this study, results suggest that an easy and clear communication to consumers for increasing knowledge and awareness with regard to spices appear a good opportunity to expand the market and consumption of spices in Italy. For example, it would be desirable to introduce spices as publicity accompanied by information about the nutraceutical content of each spice, when it is mentioned as an ingredient to flavor food, for example. This, however, often happens with regard to wine [53]. In fact, the right information can develop greater consumer awareness and therefore encourage consumption.

This study, like all the consumer studies that are carried out only in a particular geographical area, sets the limits given by the fact that it was conducted in the Sicily region (Southern Italy). It is a region of 4,948,034 inhabitants. The territory of Sicily consists almost entirely of the homonymous island, the largest island in Italy and in the Mediterranean Sea, as well as the 45th largest island in the world. Sicily is the most extensive region of Italy and the fourth in population (after Lombardy, Lazio, and Campania), and its territory is divided into 390 municipalities which in turn constituted three metropolitan cities (Palermo, Catania, and Messina) and six free municipal consortia. The island is washed to the north by the Tyrrhenian Sea, to the west by the Channel of Sicily, to the south-west by the Sea of Sicily, to the southeast by the Channel of Malta, to the east by the Ionian Sea, and to the north-east by the Strait of Messina that separates it from Calabria. The remaining part consists of the archipelagos of the Aeolian, Egadi, and Pelagie Islands and the islands of Ustica and Pantelleria.

Nevertheless, this choice was fully explained and motivated in the introduction and the high level of internationality of the island and integration between different peoples and cultures must be considered: in Italy and in Sicily, there is nowadays a high presence of immigrants [44]. Therefore, it can be considered a pilot study and it would deserve to be deepened and expanded to other Italian regions given the interesting results obtained, and hopefully, these results can be compared to those of other studies on ethnic food consumption.

## Conclusions

The analysis provided an empirical framework that offers interesting topics for reflection on the main dynamics related to the consumption of spices in Sicily. This research represents an exploratory survey on the consumption of spices in Sicily; it focused particularly on knowledge and consumption habits of consumers and on the role of product information, with the aim of understanding consumer behavior with regard to spices and their use in the daily diet. Results showed that besides the spices that Italian consumers, and specifically Sicilians, use regularly belong to their culinary culture, the so-called new/novel spices are progressively used in the regular consumption for some main reasons, such as to enhance the flavor of foods or to pursue a healthier diet, or for their beneficial properties for the body. Another interesting aspect revealed by this study is the ethnic contamination of food culture that occurred in Sicily in the last 10 years. This is very important not only from a social point of view but also and consequently from the point of view of the market and the consumption of certain foods. In fact, from a social point of view, it highlights the changes in consumer behavior that are reflected in the more frequented ethnic restaurants, driven by the curiosity to try new flavors but also to get to know the food culture of populations and people that are gradually integrating in this country and especially in this region. From the market point of view, it is important because it provides information on the change in food preferences and consequently in the possible progressive increase in consumption of the so-called new/novel spices even among Italian consumers. Finally, the results highlighted also the fundamental role that knowledge and information play in market dynamics and, how much these, can influence the demand for new products. Information on the characteristics and health benefits of these foods should be communicated to consumers in a clear and easily understandable way. The right information can develop greater awareness of the link between attributes and characteristics of the product and beneficial effects on health. Adequate communication could result in stronger purchasing intentions and loyalty, and in a greater willingness to pay by consumers.

## Data Availability

Not applicable

## Footnotes

1 Ethnic groups and foreign minorities: Bangladesh: 5,387; Sri Lanka: 3,516; Romania: 3,321; Ghana: 2,701; Philippines: 1,793; Tunisia: 1,097; Morocco: 1,058; China: 996; Mauritius: 907; Ivory Coast: 628.
